# Dating Phylogenies with Hybrid Local Molecular Clocks

**DOI:** 10.1371/journal.pone.0000879

**Published:** 2007-09-12

**Authors:** Stéphane Aris-Brosou

**Affiliations:** 1 Department of Biology, University of Ottawa, Ontario, Canada; 2 Department of Mathematics and Statistics, University of Ottawa, Ontario, Canada; University of Oxford, United Kingdom

## Abstract

**Background:**

Because rates of evolution and species divergence times cannot be estimated directly from molecular data, all current dating methods require that specific assumptions be made before inferring any divergence time. These assumptions typically bear either on rates of molecular evolution (molecular clock hypothesis, local clocks models) or on both rates and times (penalized likelihood, Bayesian methods). However, most of these assumptions can affect estimated dates, oftentimes because they underestimate large amounts of rate change.

**Principal Findings:**

A significant modification to a recently proposed ad hoc rate-smoothing algorithm is described, in which local molecular clocks are automatically placed on a phylogeny. This modification makes use of hybrid approaches that borrow from recent theoretical developments in microarray data analysis. An ad hoc integration of phylogenetic uncertainty under these local clock models is also described. The performance and accuracy of the new methods are evaluated by reanalyzing three published data sets.

**Conclusions:**

It is shown that the new maximum likelihood hybrid methods can perform better than penalized likelihood and almost as well as uncorrelated Bayesian models. However, the new methods still tend to underestimate the actual amount of rate change. This work demonstrates the difficulty of estimating divergence times using local molecular clocks.

## Introduction

Estimating divergence times from molecular data is a special statistical endeavor, as the parameters of interest cannot be directly estimated from molecular sequences: only distances between pairs of sequences or site likelihood values can be estimated. Such distances are measured in terms of the expected number of changes per site along the molecule (DNA, RNA or protein). This is equivalent to taking the product of a rate of molecular evolution and of a time duration, two quantities of significant biological interest. Unfortunately, neither rates nor times are identifiable parameters. As a result, estimating divergence times demands that special assumptions be posited. To this end, four approaches are commonly used in a model-based framework.

Until recently, the approach of choice to estimating divergence times was to assume the molecular clock [1], i.e. that rates are constant over the entire history of the genes under study. Under this hypothesis, branch lengths are directly proportional to time, and the common evolutionary rate is determined by the placement of at least one fossil calibration point on the phylogeny. However, the development of specific hypothesis tests (e.g., ref. [2]) showed that the molecular clock is too often untenable [3].

A second approach to estimating times while relaxing the above assumption of the clock is to posit several local clocks. The incentive here is that the clock hypothesis is likely to hold for closely related species [4]–[6]. Local clock models were recently extended to incorporate multiple calibration points and multiple genes [7]. However, one important shortcoming of this approach is that the placement of the different local molecular clocks on the tree is left to the user's discretion. I show below how this can affect inference.

A third and alternative approach, pioneered by Sanderson, is to consider that rates of evolution evolve from ancestral rates. This implies that rates of evolution are (auto-) correlated [8]. More recently, Sanderson [9] proposed to use an approach that maximizes a penalized likelihood *p*(*X*|***r***.***t***)–*λ* Φ(***r***), where *X* is the data alignment and ***r***.***t*** represents the branch lengths, i.e. the product of rates ***r*** and times ***t***. The penalty function Φ(***r***) is chosen in such a way that it is large when rates vary rapidly over small regions of the tree; the smoothing parameter *λ* is estimated by cross-validation [10].

Expanding on this idea, Thorne and coworkers [11] proposed a Bayesian approach for estimating divergence times. This fourth approach relies on prior models of speciation and of autocorrelated rate change. These prior models are in fact equivalent to a particular penalty function in Sanderson's approach [10], so that both of these approaches are often referred to as “regularization methods” in the literature of supervised learning. Although prior distributions and implementation details vary, the Bayesian approach and penalized likelihood both have in common to smooth or minimize rate variation over evolutionary time by means of an autocorrelated process. Because this can lead to inaccurate dates when rates vary extensively [12], a class of prior models of uncorrelated rate change were recently described [13]. A different Bayesian approach has also been described [14], where rate change events are modeled as point processes on a phylogeny. This approach does have the potential of accommodating rapid rate variation, but the specific model used in that study was shown to be overparameterized [15]. Another potential issue, this time common to all Bayesian approaches, is the need to specify prior assumptions: some, such as prior distributions on the speciation process, are potentially overly informative [16], [17], while others, such as the root rate, can be difficult to set [18]. Bayesian approaches can also be computationally demanding.

An alternative to regularization methods is to reconsider local clocks. These models are expected to be able to accommodate high levels of rate heterogeneity, and therefore to perform better. However, as noted above, there are two difficulties with local clock models. First, the number of local clocks has to be chosen and second, local molecular clocks must be placed on a given phylogeny. Yang [19] suggested a clever hybrid algorithm to address the second difficulty. This approach, called ad hoc rate-smoothing (AHRS), involves three steps: (i) branch lengths are estimated for each gene on a pre-specified common gene tree without the restriction of the clock; (ii) first, initial branch-specific rates of evolution are approximated based on a parametric smoothing approach that minimizes rate variation; then, an ad hoc clustering algorithm is used to group these branch-specific rates into clusters that constitute the local clocks; (iii) divergence times are finally estimated by maximum likelihood following [7]. Note that step (ii) actually consists of two distinct stages. Confidence intervals can be obtained following standard asymptotic methods either based on a normal approximation of the likelihood surface or on the support curve [20]. While the AHRS algorithm makes it possible to place the local molecular clocks on a phylogeny, the number of clocks is still left to users' discretion–the first “difficulty” highlighted above.

Here I present four new ad hoc methods that improve on Yang's AHRS algorithm. The methods described below essentially modify one or both stages of step (ii) of the original AHRS algorithm. The four new methods borrow from recent advancements in the field of statistical analysis of microarray data. As these new methods all rely on maximum likelihood estimation, they are apparently immune to the common criticisms that Bayesian approaches occasionally draw in particular with respect to the choice of a prior [2], [21], and that they are computationally efficient. I compare these methods with penalized likelihood [9] and with a Bayesian model of uncorrelated rate change [13], suggest a way to integrate phylogenetic uncertainty, and evaluate the performance and accuracy of these methods on three previously published data sets. The source code and precompiled binaries for common 64-bit architectures (x86, ultra-sparc and PPC) are available at http://aix1.uottawa.ca/∼sarisbro.

## Methods

### First improvements with k-means and k-medoids

In the original AHRS algorithm, branch lengths are first estimated by maximum likelihood (ML) without assuming the clock–see step (i) above. The likelihood surface is then approximated by a multivariate normal distribution *N* (***B***,***Σ***) centered on the ML estimates (MLEs) ***B*** of the branch lengths. The variance-covariace matrix ***Σ*** is approximated by the diagonal variance matrix of the estimates of the branch lengths–note that these variances were previously estimated during the optimization in step (i). This approximation of ***Σ*** assumes that there is no correlation between these MLEs, which is computationally faster than the approximation based on the full Hessian matrix as e.g. in [11], [19], [22]. A Brownian motion model [11], [22] is then used to estimate by ML the initial branch-specific rates at beginning of step (ii) as in [19]. Following this, the AHRS algorithm uses an ad hoc clustering scheme to group similar rates into local clocks. Briefly, the interval bounded by the smallest (*m*) and the largest (*M*) initial rate estimates is divided up into *k* groups; the coordinates of the cutoff points are solely a function of *m*, *M* and *k*. Here, I reused all these steps and modified the source code of PAML ver. 3.14 [23] to incorporate two true clustering schemes: the k-means algorithm [24] and the k-medoids algorithm [25]. The k-means algorithm was run with 1,000 random starts to find the solution with lowest within-cluster sums of squared dissimilarities. Unlike k-means, k-medoids minimizes a sum of dissimilarities and is therefore expected to be more robust. Both algorithms were used as implemented in R (cran.r-project.org, in the stat and cluster packages). R was called externally from PAML.

The estimation of the number of clocks in a standard likelihood framework is not easy, as the models are generally not nested and are typically derived from the data. Instead, the number of clocks was in a first approach selected using an idea based on the gap statistic [26]: if there were actually k* clocks on the tree, then for *k*<k*, each partition calculated by the algorithm would contain a subset of the actual groups of branches, and the algorithm would not assign branches as it “should”. Therefore, the likelihood should increase substantially each time *k* is increased. Now for *k*>k*, one of the calculated clusters would partition one or more of the actual groups, which should tend to provide smaller likelihood increases. Indeed, splitting a genuine group is not expected to increase the likelihood more than partitioning the union of two clearly separated groups. As a result, when the log-likelihood is plotted as a function of *k*, there should be a kink in the plot. A natural estimate of k* can be taken at this value, that is, at the largest gap in likelihood values between two consecutive values of *k*.

### Automatic estimation of the number of clocks with silhouettes and HOPACH

Our loose implementation of the gap statistic does not allow for a rigorous estimation of the number of local clocks. However, the k-medoids algorithm offers the possibility to select the number of clusters according to the Median Split Silhouette or MSS [27]. Based on normalized differences of dissimilarities, silhouettes measure the homogeneity of each element when grouped in a cluster compared to the situation where this element is left out. The median of these measures taken over all elements and over all clusters is a measure of the homogeneity of each cluster, and therefore constitutes an objective function to find the most appropriate number of clusters.

In cases where branch-specific rates of evolution vary rapidly, MSS may not be aggressive enough to find small clusters. A more recent method, the Hierarchical Ordered Partitioning and Collapsing Hybrid or HOPACH [28] aims at finding such small clusters. Although HOPACH makes use of a partitioning method based on k-medoids, it is actually a hierarchical method. As such, it involves the construction of a tree of clusters where the “root” of the hierarchy contains all the elements (here, all the branch-specific rates) and where each leaf contains one single element. HOPACH is an iterative algorithm that starts with all the elements at the root node of the hierarchy, and that cycles through partitioning, ordering and collapsing as follows. At each level of the hierarchy, the partitioning step uses k-medoids and silhouettes to determine the clusters, which are then ordered according to the dissimilarity between their medoids or centers. Below root-level, pairs of clusters are collapsed if and only if doing so improves the median split silhouette. The algorithm stops when each node contains at most two elements, or when a maximum number of levels is reached, whichever comes first. Because the elements contained at each level of the hierarchy are labeled with integers, this upper bound is set to 16 levels in the current implementation of the R package hopach (ver. 1.4.0) to avoid overflows.

However, for identifiability reasons, we cannot afford as many local clocks as there are branches in the tree. Under the multiple gene models used here, where branch-specific rates are estimated separately and independently for each of the *g* genes [7], there is a total of *kg* rate parameters when *k* local clocks are used. With *n* sequences and *c* calibration points, we want to estimate *n*–1–*c* divergence times, so that there is a total of *n*–1–*c*+*kg* parameters to estimate. The total number of identifiable parameters is (2*n*–3) *g*, so that *k*, the number of local clocks, should not exceed *U* = 2*n*–3–(*n*–1–*c*)/*g*. For this reason, the maximum number of clusters returned by MSS and HOPACH was limited to int(*U*), the integer part of *U*. Yet, even when *k*<*U*, certain placements of the local clocks make it impossible to identify all model parameters–see [6] for a practical example. To help guarantee against this serious issue, convergence of our procedures was checked by running each likelihood analysis a thousand times starting from random initial parameters.

### Improved initial branch-specific rates

Like the original AHRS algorithm, the new ad hoc methods presented here all critically rely on the initial ML estimation of the branch-specific rates of evolution (step (i) of AHRS). These rates are the starting point of the clustering procedures used to determine both the placement and the number of local clocks over the tree. Instead of estimating these initial branch-specific rates with Brownian motion model, these initial rates were also obtained with a Bayesian procedure, before being clustered into local clocks. Here I used the Bayesian approach detailed below to determine these rates under an uncorrelated lognormal model of rate change [13].

### Ad hoc integration of phylogenetic uncertainty

The objective here is not to implement a Bayesian version of local clock models, one that would integrate over tree topologies. However, I indicate two approximations for dealing with phylogenetic uncertainty with these models. The most appropriate ad hoc procedure would follow two steps. First, draw samples from the posterior distribution *p*(*τ* | *X*) = ∫_Θ_ *p*(*τ*, ***θ*** | *X*) *d* ***θ*** , where *X* represents the aligned data, *τ* is the tree topology and ***θ*** is a vector of nuisance parameters, typically the branch lengths and the parameters of the substitution model. The model should not enforce the clock. If the sampled trees are not rooted they should be rooted before moving on to the next step. Second, evaluate divergence times for all the samples drawn during the previous step. The “posterior” estimate of the divergence time at a given bipartition is then estimated as the average time estimated for each of the sampled trees, when this bipartition exists. The last restriction is equivalent to enforcing a number of monophyletic relationships. This second step is the most computationally demanding as it requires performing ML optimizations for each sample drawn from the Markov chain in step one. A similar two-step approach was previously used to infer the ancestral state of morphological characters in the presence of phylogenetic uncertainty [29]–see [30] for a recent application.

Rather than following this direction here, I used an even more ad hoc but faster procedure. Trees were sampled from their posterior distribution with MrBayes ver. 3.1.2 [31] with tempering (four chains). Four such independent samplers were run for five million steps with thinning of 1,000 and a burn-in of a million. Divergence times were then estimated with the local clock models described above for each of the topologies contained in the 95% credibility set: the original AHRS, the k-means and the k-medoids algorithms (the number of clocks was estimated with the gap method for all these three), MSS and HOPACH. As above, the ML optimization procedures were repeated (only 20 times here) to check convergence and identifiability.

### Data sets and models of evolution

I evaluated the performance of the new methods on three previously published data sets. The first one is a mouse lemur data set repeatedly used when developing dating methods [7], [19]. This data set contains the concatenated sequences of two mitochondrial genes, COX-II and CYT-b, sampled over a total of 35 mammalian species. The objective of the original study [7] was to estimate divergence times of mouse lemurs (genus: Microcebus), the world's smallest primates, endemic to Madagascar. The data contained 604 codons and the analyses assumed the one-ratio model [32]. All model parameters were estimated by ML, except equilibrium frequencies, calculated assuming the F3×4 model. Seven calibration points were used to estimate divergence times as in [19]–seeSupp orting Information Figure S1. This data set was used here to test whether the new clustering methods can improve on some of the shortcomings of the original AHRS method.

The accuracy of the new methods was then evaluated by reanalyzing a sea urchin data set for which the fossil record is considered excellent [33]. This data set consists of three rRNA genes, one mitochondrial (16S large subunit) and two nuclear (18S small subunit and 28S large subunit) genes. I used the unpartitioned alignment of 3,331bp for 28 species as the original authors (see Table 2 in [33]). Because the GTR+Γ_6_+I substitution model [34], [35] used in [33] is not available in PAML's local clock implementation, I used HKY+Γ_5_ [36]; five rate categories were used for computational reasons. Unlike the quite unfavorable calibrating situation represented by the mouse lemur data, the four calibration points used by [33] and here are quite evenly distributed over the tree, and vary from 55 to 210 MY of age.

A third data set was used to assess the impact of uncertainty over tree topologies when estimating species divergence times. I reused the alignment originally assembled by [37] and reanalyzed by [13]. The alignment contains five protein-coding genes (APOB, RAG1, IRBP, vWF, and BRCA1) for 24 taxa. All 3,772 positions were analyzed as one single partition. Four calibration points were used as in [13]. The Bayesian model placed normal priors of variance 5 MY on the three internal nodes and constrained the root node around 145 MY with an exponential prior as in [13]. The substitution model was again set to HKY+Γ_5_. This substitution model is slightly simpler than the one used by [13], as the dating approaches described here do not allow for invariant sites or more complicated models such as GTR.

### Comparison with other dating approaches

The methods described above were compared with two existing dating methods: penalized likelihood and an uncorrelated Bayesian model of rate change. Simulations are often considered as an important step in checking the validity of a method, as they permit to check that a method returns the correct answer, at least under simple scenarios. However, because real sequence data rarely evolve following simple models and because a given simulation model can bias the results towards a particular direction [38], [39], I resorted to a different approach here, and used the data set from [33] for which the actual fossil record is unusually complete. For this specific data set, it was found [33] that the penalized likelihood approach [9] seemed to perform better than both nonparametric rate smoothing [8] and a Bayesian approach [11]. As the objective here was not to reevaluate all existing dating methods against the ones presented here, I focused on the “best” method identified by [33], namely, penalized likelihood (PL). I recalculated date estimates using PL with r8s ver.1.70 [40] following two approaches: PL^(CV)^ and PL^(SSD)^, where CV and SSD stand for cross-validation and sum of the squared distances, respectively. The first, PL^(CV)^, used cross-validation [9]; this is usually the only method that can be used when the actual dates are unknown. The accuracy of PL^(CV)^ was determined with the second approach: PL^(SSD)^. When the actual divergence times are known for all the nodes on the tree, as with the sea urchin data set, it is possible to select the smoothing parameter *λ* that best fits the actual fossil dates. To this effect, *λ* was varied from 0 to 10^5^ by increments of 10. The “best fit” was quantitatively measured by the sum of the squared distances (SSD) between each time estimate and its corresponding fossil age. The minimum SSD corresponds to the set of ages that is closest to the actual fossil dates. Note that by taking the square of these distances, this criterion penalizes methods that have large discrepancies between time estimates and fossil ages. This SSD criterion made it possible to assess the performance of PL and of the new ad hoc methods. The number of clocks *k* under k-medoids was optimized in a similar way.

The uncorrelated Bayesian model of rate change implemented in BEAST ver. 1.4.4 [13] was used to estimate divergence times and to determine rates. The substitution models matched those used for the ML analyses. I assumed the uncorrelated lognormal (UCLN) prior model of rate change, and a Yule or “pure-birth” prior process to model speciation. For each data set, two samplers were run independently with BEAST, each for 50 million steps with a thinning of 1,000. Five million steps were discarded as burn-in. Convergence of the Bayesian analyses, including the MrBayes runs for the ad hoc integration of phylogeny, was checked with Tracer (tree.bio.ed.ac.uk /software/tracer).

## Results

### Mouse lemur data: the benchmark data

**Figure 1:**
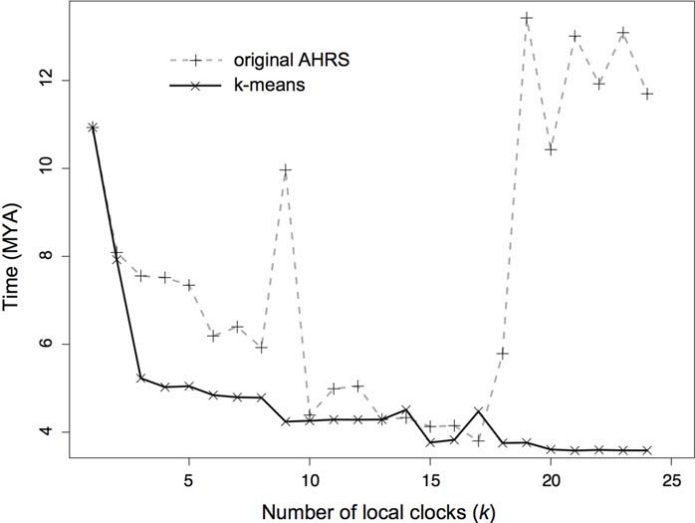
Effect of the number of local clocks on the age of a node far from all calibration points. Times were estimated using either the original AHRS algorithm or k-means and are given in million years ago (MYA). The focal node is the split at the origin of the mouse lemurs (genus Microcebus).

The first important question is to what extent the number *k* of local clocks can affect the estimation of divergence times. The divergence time of some nodes can be relatively insensitive to *k*. For instance, with the k-means clustering algorithm, the estimated dates for the hominoid divergence (node 59 in Table 1) vary from 13.5 million years (MY) to 13 MY when the number of local clocks *k* is varied from one, which is the molecular clock assumption, to six. But the age of some other nodes can be more difficult to estimate. Such is the case with Microcebus diversification (node 45 in Table 1). Figure 1 shows that irrespective of the clustering algorithm used, the age of this diversification varies between about 8 MY with two clocks, to an age less than half this figure with more than 15 clocks. It is therefore quite important to have a means of estimating the most appropriate number of clocks.

**Table 1:** Maximum likelihood estimates of divergence times for the mouse lemur data under nucleotide and codon models.

node #	split	original	kmeans	kmedoids	HOPACH	MSS
38	Strepsirrhine	56.3	50.1	50.3	50.6	62.0
39	lemuriform	47.3	43.8	44.2	44.5	55.0
43	Cheirogaleidae	17.7	14.9	12.9	13.8	29.8
**45**	**mouse lemurs**	**7.6**	**5.2**	**4.5**	**4.9**	**12.0**
46	northern clade	6.0	4.1	3.5	3.9	9.6
52	southern clade	5.3	3.7	3.1	3.4	8.9
53	Lemuridae	21.4	18.9	19.1	19.2	24.1
56	lorisiform	31.6	25.9	25.9	26.0	33.3
57	anthropoid	58.8	59.6	59.6	59.6	60.7
59	hominoid	13.2	13.9	13.9	13.9	15.8
**61**	**human/chimp**	**7.0**	**7.3**	**7.3**	**7.3**	**7.0**
64	dog/bear	40.9	45.4	45.4	45.4	41.4
lnL		−25053.27	−25060.11	−25060.35	−25060.54	−24869.79

Times are in million years ago.

Notes–lnL: log-likelihood.

A second point should be made here. Figure 1 also shows that local molecular clocks can experience some identifiability issues for some placements of the clocks (e.g., at *k* = 9) and further difficulties when the number of clocks becomes too large (over 17 clocks in the case of this data set). A careful examination revealed that when the number of clocks increases, the ad hoc clustering algorithm used in AHRS eventually returns a number of empty clusters. This is because once the lower and upper bounds of the initial rates are estimated (during step (i) of AHRS), cluster boundaries are set deterministically, disregarding the distribution of these initial rates. Standard clustering algorithms such as k-means appear to resolve this problem (Figure 1), at least in this specific example.

**Figure 2:**
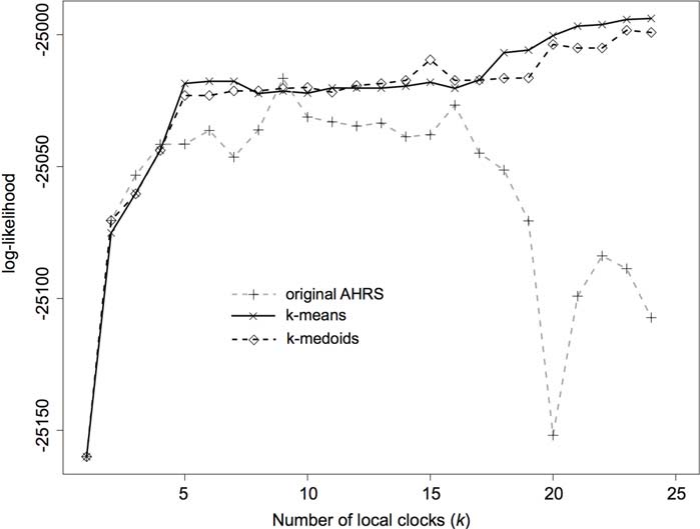
Maximum likelihood score as a function of the number of clocks.

With a proper clustering algorithm, how difficult is it to find the appropriate number of clocks? Another important observation from Figure 1 is that implementing two or three local clocks affects age estimates quite dramatically. These estimates range from 10.93 MY under a global clock to 7.5 MY with three local clocks. With more than three clocks, age estimates change less rapidly. Figure 2 plots the log-likelihood under a codon model as a function of *k*. The application of the idea behind the gap statistic to this figure suggests that the appropriate number of clocks is three for this data set. As a more rigorous approach than our implementation of the gap statistic, HOPACH finds the same number of local clocks (k* = 3) and distributes them similarly to k-medoids over the phylogeny (Supporting Information Figure S1). The likelihood scores obtained under the codon model (Table 1) are slightly smaller than in [19] as our procedures consistently selected three local clocks instead of four [19]. Both k-medoids and HOPACH suggest similar age estimates (Table 1), with most notably a recent diversification (<5 MY) of the genus Microcebus. MSS on the other hand suggests one additional clock, with a fragmented partition scheme over the phylogeny (Supporting Information Figure S1) and a dramatically different picture of the diversification of mouse lemurs, suggesting that these would date back to 12 MY ago (Table 1). Because of a recently found 38–42 MY fossil for the ancestor of loris and galago [41], Yang suggested that an age of 5 MY is too young for the mouse lemur divergence, and found an age of 11.4 MY only after including the additional information about this extra fossil in the analysis [19]. Our MSS results under a codon model (divergence at 12.0 MY) may suggest that sufficient information exists in the data without this additional calibration point. However, this result is not confirmed by the use of MSS under a nucleotide model (F84+Γ_5_: divergence at 6.6 MY). This difference is likely to be due to the relative number of clocks estimated under each model for a given data set: under a codon substitution model, MSS has here the largest number of clocks (Supporting Information Figure S1), while under a nucleotide substitution model, it is HOPACH that has the largest k* (Supporting Information Figure S2). This raises the important question about the accuracy of these approaches.

### Sea urchin data: accuracy against the fossil record

When only four calibration points are used, MSS had the second best accuracy (smallest SSD in Table 2; disregarding the last column for now). However, when all the fossil dates are considered, PL^(SSD)^ and k-medoids optimized for *k* show that even MSS tends to underestimate rate change, and hence the number of clocks. This suggests that it might be difficult to accommodate in a statistically sound manner actual amounts of rate variation in real data with these maximum likelihood methods.

**Table 2:** Comparison of divergence times from fossils with those estimated from molecular data and four calibration points for the sea urchin data.

Smith nodes¶	Fossil age †	PL^(CV)^ ¥	PL^(SSD) ^(λ = 100)	k-med¥ k = 3	k-med k = 12	HOPACH¥	MSS¥	MSS+UCLN¥	UCLN¥
root	255	255	255	255	255	255	255	255	255
1	220	189	190	255	225	255	255	255	233
2	210	187	189	245	210	239	241	225	246
3	210	163	171	245	210	239	241	225	216
4‡	210	135	150	210	210	210	210	210	210
5	200	175	179	160	173	160	160	160	213
6	185	164	173	131	173	129	125	147	188
7	175	148	161	131	173	129	125	147	172
8	105	124	136	117	156	107	111	121	137
9	105	97	105	94	119	89	90	90	106
10	85	38	73	71	119	43	73	36	41
11‡	55	86	90	55	55	55	55	55	89
12	55	37	35	36	39	38	33	38	40
13	30	74	75	51	46	51	50	51	68
14	170	144	157	119	173	129	125	147	168
15‡	95	95	95	95	95	95	95	95	94
16	80	68	78	33	68	47	50	58	67
17	55	50	61	21	50	30	32	39	60
18	45	29	39	0	0	0	0	0	32
19	40	33	41	11	29	16	17	21	36
20	195	168	170	160	160	160	160	160	179
21‡	160	160	160	160	160	160	160	160	159
22	100	104	111	113	157	92	97	116	129
25	100	93	98	99	120	77	85	98	128
26	20	25	29	32	35	24	26	36	30
23	30	62	75	46	65	45	36	55	44
24	25	58	70	46	65	45	36	55	59
SSD	na	21056	17125	22458	15615	20968	19796	16036	11192

For k-medoids (k-med), the optimal number of local clock is at k* = 3; the minimum sum of squared distance (SSD) is at k = 12.

Notes–From Smith *et al*. (2006): Fig. 5 (^¶^) and Table 2 (^†^). ^¥^ Naïve approaches, for which knowledge of the actual fossil dates is not taken into account to estimate divergence dates. ^‡^ Calibration points (set as minimum ages for PL); root age is fixed. na: not applicable. PL^(CV)^ contains age estimates obtained by penalized likelihood with cross-validation; with PL^(SSD)^, the penalty is selected by minimizing SSD.

One potential reason why rate change is underestimated might be because of the way initial branch-specific rates are estimated. Recall that these initial estimates come from an autocorrelated Brownian motion model (see above) that minimizes rate change over the tree. To test the impact of such a procedure on our hybrid local clock procedures, I first obtained branch-specific rates under an uncorrelated model of rate change, and then fed these rates to the hybrid methods. These initial rates were estimated with BEAST under the UCLN model by running two MCMC samplers independently. The two BEAST runs proved very similar, so their results were combined. The effective size of the samples taken from the target distribution was 51,300. The posterior mean rates over the maximum a posteriori tree were used as starting point under MSS (MSS+UCLN in Table 2). Posterior point estimates of divergence times (mean of the marginal distributions) are also reported in the last column of Table 2.

SSD results show that for this data set, the use of Bayesian estimates of rates improves our MSS hybrid procedure. As expected, the SSD score obtained under MSS+UCLN (16,036) shows that this latter procedure still underestimates rate change, as k-medoids had a higher score (15,615) with *k* = 12. Finally, the UCLN results show that, quite as expected again, uncorrelated models of rate change performed better than any other approaches considered here.

### Marsupial data: ad hoc dating with uncertain phylogenies

The analyses with the hybrid ML local clock models all assumed that the tree topology is known, which is not always a realistic assumption. To evaluate the impact of this assumption on date estimates, I reanalyzed the recently published marsupial data set. Date estimates were first obtained with BEAST. The effective size sampled from the target distribution of UCLN was 29,580. Point estimates of divergence times (mean of the marginal distributions) are reported in the first column of Table 3.

**Table 3:** Effect of neglecting phylogenetic uncertainty on estimates of divergence times under local clock models for the marsupial data.

split	MSS: MAP tree	MSS: 95% CS	HOPACH: MAP tree	HOPACH: 95% CS	UCLN (BEAST)
*Sminthopsis/Phascogale*	24.76	24.77	23.55	23.71	23.96
*Sminthopsis/Echymipera*	57.92	57.87	55.55	55.49	56.06
*Echymipera/Perameles*	12.39	12.39	11.09	11.16	13.09
*Notoryctes/Sminthopsis*	61.05	60.96	59.07	59.10	59.72
*Dendrolagus/Pseudocheiridae*	35.07	35.26	45.12	44.19	33.26
*Dendrolagus/Phalanger*	37.92	37.92	48.52	47.73	39.58
*Phalanger/Vombatus*	40.46	40.61	50.67	50.09	46.25
*Vombatus/Phascolarctos*	26.27	26.35	33.77	33.01	31.29
*Vombatus/Dromiciops*	55.80	56.52	62.54	62.43	61.46
*Dasyurus/Rhyncholestes*	73.23	72.20	76.74	74.30	77.67
*Caenolestes/Rhyncholestes*	10.66	10.49	13.88	11.75	13.71
*Equus/Ceratomorpha*	46.29	46.29	43.65	43.44	43.20
*Cynocephalus/Leporidae*	67.24	67.27	63.71	64.40	64.74
*Cynocephalus/Ceratomorpha*	80.23	80.25	76.66	76.81	77.54
*Ceratomorpha/Bradypus*	90.88	90.88	92.15	92.05	87.94
*Bradypus/Sirenia*	87.07	87.05	88.94	88.81	80.87
SSD	202.28	203.75	338.07	304.87	0.00

Results for local clock models, MSS and HOPACH, are given both for the maximum a posteriori (MAP) tree and as a posterior probability weighted average over the 95% credibility set (CS) of topologies sampled from the appropriate target distribution. The uncorrelated models from BEAST (UCLN: uncorrelated lognormal) integrate over tree topologies, branch lengths and parameters of the substitution model. Times are in million years ago. SSD are computed with respect to UCLN (baseline).

I compared this uncorrelated Bayesian model with the local clock procedures with ad hoc integration of phylogenetic uncertainty. With MrBayes, the maximum a posteriori (MAP) tree had a posterior probability of .43, and the 95% credibility set included a total of nine tree topologies. The time estimates presented in Table 3 represent average estimates, weighted by the posterior probability of the tree topologies. Credibility intervals for time estimates can easily be obtained when times are estimated for each sample drawn from the Markov chain, but not under this latter ad hoc implementation of integrated local clocks. For this reason, no standard errors can be reported in Table 3.

How do these Bayesian time estimates compare with those based on ML local clock? Two conclusions can be drawn from Table 3. First, averaging over phylogenetic uncertainty had little impact on the estimates of divergence times, at least in this example: under a given method, only minor differences were found between estimates based on the MAP tree and ad hoc integration over the 95% credibility set of topologies. Second, the largest differences occur between MSS and HOPACH, the uncorrelated Bayesian approach giving somewhat intermediate results. This is in particular true for two splits, Dendrolagus/Phalanger and Phalanger/Vombatus, which can exhibit an age difference of up to 10 MY between the two methods. These two nodes have a posterior probability of one, so that phylogenetic uncertainty is not responsible for this difference in age estimates. SSD suggests that MSS give results that are the closest to UCLN.

## Discussion

As always, it is advisable to use several methods to estimate a parameter of interest, such as divergence times between different species. Compared to regularization methods [9], [11], local molecular clocks potentially have the advantage of accommodating rapid rate variation along lineages, without incurring the computational overhead of Bayesian uncorrelated methods [13]. The methods presented here constitute a significant improvement of the AHRS algorithm for the automatic placement of local molecular clocks by providing researchers with a means to determine how many clocks should be used to analyze their data. The results presented here show that these ML local clocks, based on hybrid statistical approaches, constitute a computationally quick alternative to Bayesian methods, without requiring the setting of reasonable prior distributions. Our results also demonstrate that the choice of a specific algorithm can have a dramatic impact on date estimates.

These hybrid methods however appear to present four potential limitations. The first one, common to both the original AHRS algorithm and these new hybrid methods, is the reliance on the initial estimation of approximate rates by ML in step (i) of the procedure [19]. I showed here that better initial estimates of these rates can help improve the accuracy of the divergence dates estimated. But because these improved estimates were based on an MCMC sampler, the speed advantage of the hybrid methods disappears. A second important limitation of these hybrid methods is their underestimating rate change. The underestimation of rate change is not a surprise, since this is precisely the idea driving the use of local clocks: reducing the extent of rate variation. Third, all possible placements of local clocks on a tree do not lead to identifiable parameters [6]. In our implementation, this is still an important issue as no rigorous identifiability check is currently implemented. Finally, confidence intervals, not estimated here, are expected to be underestimated as uncertainty about model parameters is disregarded. Confidence intervals will also be difficult to obtain when the topology is integrated following the ad hoc procedure outlined above. Bayes empirical Bayes strategies, as recently employed to detect sites under adaptive evolution [42], could prove valuable for these ad hoc dating methods. As noted in [20], other limitations of the likelihood methods exist, in particular with respect to the incorporation of uncertainties about calibration points into an analysis; these limitations are however naturally dealt within a Bayesian framework [20].

## Supporting Information

Figure S1 Maximum likelihood estimates of divergence times for the codon data partitioned according to the estimated local clock models. Times were estimated using (A) k-medoids with the gap statistic, (B) Median Silhouette Splits (MSS) and (C) Hierarchical Ordered Partitioning and Collapsing Hybrid (HOPACH) and are given in million years ago (MYA). Filled circles indicate the seven calibration points on the trees scaled to time (A–C); the other trees (D–F) are scaled to the expected number of substitutions per codon.
(0.45 MB PDF)

Figure S2 Maximum likelihood estimates of divergence times for the nucleotide data partitioned according to the three codon positions and the estimated local clock models. Times were estimated using k-medoids with the gap statistic, Median Silhouette Splits (MSS) and Hierarchical Ordered Partitioning and Collapsing Hybrid (HOPACH) and are given in million years ago (MYA). Filled circles indicate the seven calibration points on the trees scaled to time (leftmost column); the other trees are scaled to the expected number of substitutions per nucleotide site for the partitions over the three codon positions.
(0.38 MB PDF)
