# Supplementary figures and images for: Dating Phylogenies with Hybrid Local Molecular Clocks
Source: PLoS One. 2007 Sep 12;2(9):e879. doi: 10.1371/journal.pone.0000879 (PMC1964517; doi:10.1371/journal.pone.0000879)

A

k-medoids + gap

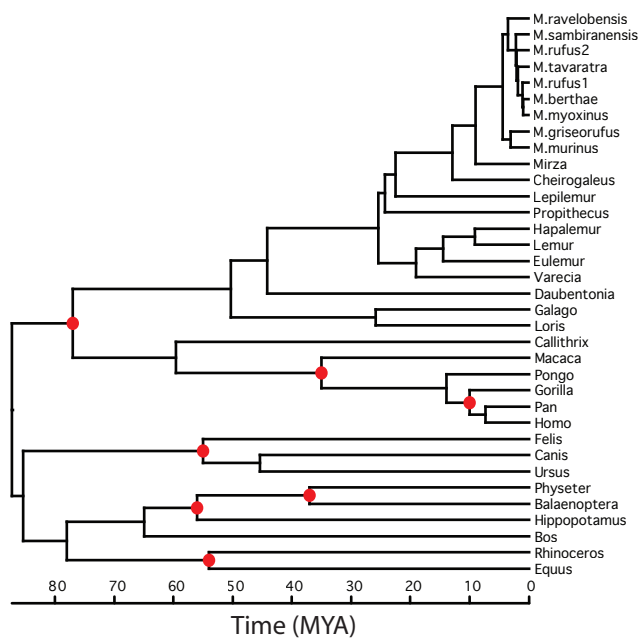

B

MSS

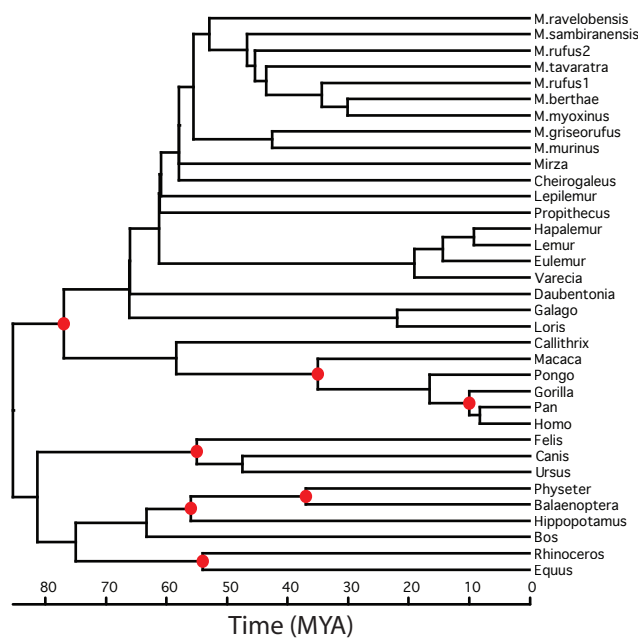

C

HOPACH

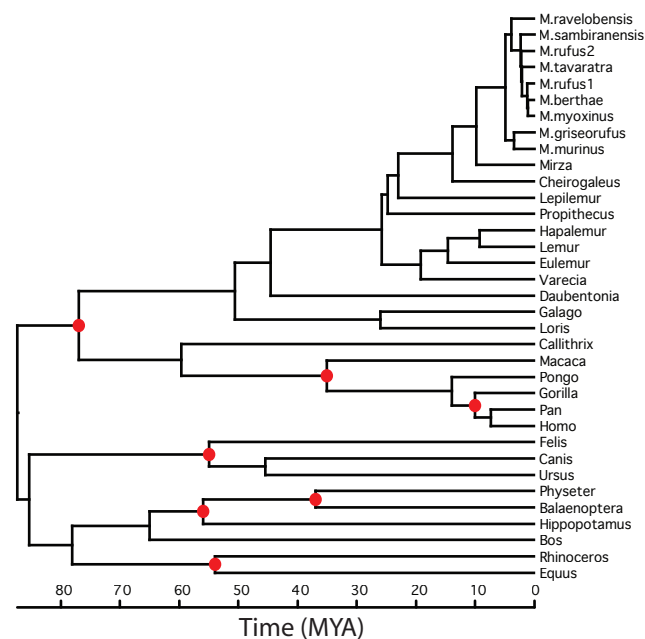

D

 $k^* = 3$ 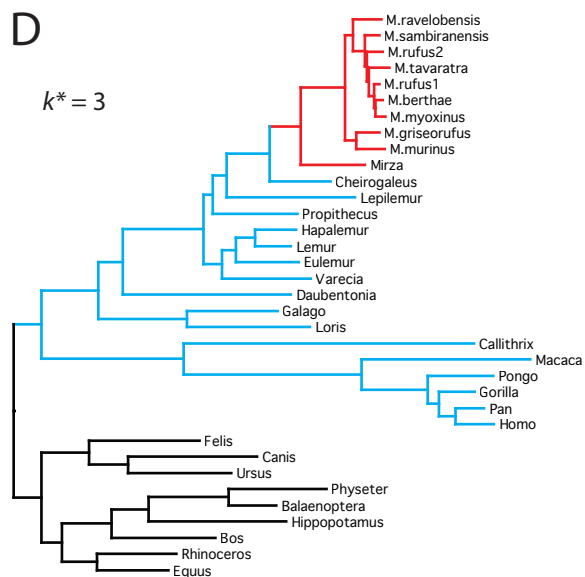

E

 $k^* = 4$ 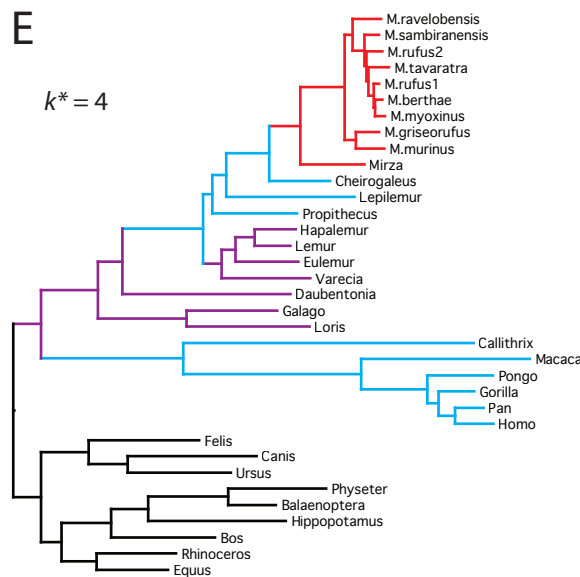

F

 $k^* = 3$ 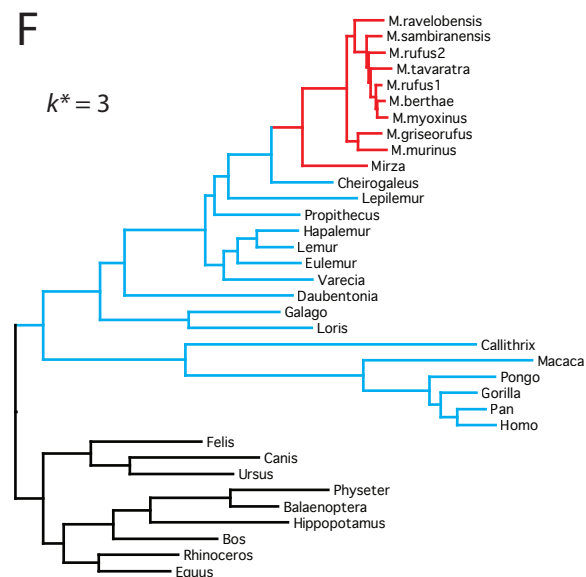

0.1

Supplement: Figure S1 — Maximum likelihood estimates of divergence times for the codon data partitioned according to the estimated local clock models. Times were estimated using (A) k-medoids with the gap statistic, (B) Median Silhouette Splits (MSS) and (C) Hierarchical Ordered Partitioning and Collapsing Hybrid (HOPACH) and are given in million years ago (MYA). Filled circles indicate the seven calibration points on the trees scaled to time (A–C); the other trees (D–F) are scaled to the expected number of substitutions per codon. (0.45 MB PDF) [file pone.0000879.s001.pdf]
