# Supplementary material for: Dating Phylogenies with Hybrid Local Molecular Clocks
Source: PLoS One. 2007 Sep 12;2(9):e879. doi: 10.1371/journal.pone.0000879 (PMC1964517; doi:10.1371/journal.pone.0000879)
Supplement: Figure S2 — Maximum likelihood estimates of divergence times for the nucleotide data partitioned according to the three codon positions and the estimated local clock models. Times were estimated using k-medoids with the gap statistic, Median Silhouette Splits (MSS) and Hierarchical Ordered Partitioning and Collapsing Hybrid (HOPACH) and are given in million years ago (MYA). Filled circles indicate the seven calibration points on the trees scaled to time (leftmost column); the other trees are scaled to the expected number of substitutions per nucleotide site for the partitions over the three codon positions. (0.38 MB PDF) [file pone.0000879.s002.pdf]

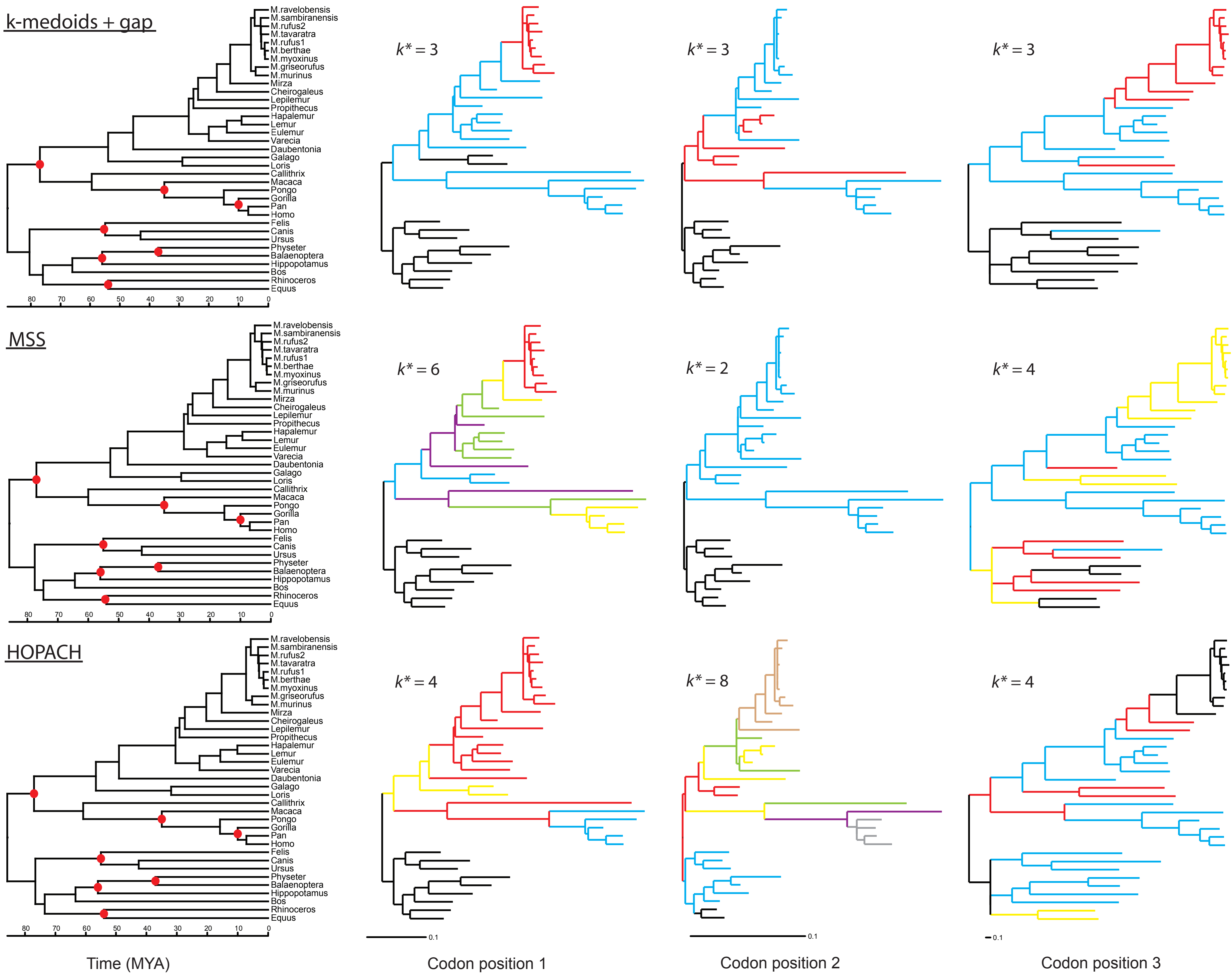

k-medoids + gap
MSS
HOPACH
M.ravelobensis
M.sambiranensis
M.rufus2
M.tavaratra
M.rufus1
M.berthae
M.myoxinus
M.griseorufus
M.murinus
Mirza
Cheirogaleus
Lepilemur
Propithecus
Hapalemur
Lemur
Eulemur
Varecia
Daubentonia
Galago
Loris
Callithrix
Macaca
Pongo
Gorilla
Pan
Homo
Felis
Canis
Ursus
Physeter
Balaenoptera
Hippopotamus
Bos
Rhinoceros
Equus
80 70 60 50 40 30 20 10 0
$k* = 3$
$k* = 3$
$k* = 3$
$k* = 6$
$k* = 2$
$k* = 4$
$k* = 4$
$k* = 8$
$k* = 4$
0.1
0.1
0.1
Time (MYA)
Codon position 1
Codon position 2
Codon position 3
